# Verification of setup errors in external beam radiation therapy using electronic portal imaging

**DOI:** 10.4103/0971-6203.41192

**Published:** 2008

**Authors:** K. Krishna Murthy, Zakiya Al-Rahbi, S. S. Sivakumar, C. A. Davis, R. Ravichandran, Kamal El Ghamrawy

**Affiliations:** Department of Radiotherapy, National Oncology Center, the Royal Hospital, Muscat, Sultanate of Oman

**Keywords:** Anatomy matching software, electronic portal imaging device, immobilization casts, setup error, tumor control probability

## Abstract

The objective of this study was to conduct an audit on QA aspects of treatment delivery by the verification of the treatment fields′ position on different days to document the efficiency of immobilization methods and reproducibility of treatment. A retrospective study was carried out on 60 patients, each 20 treated for head and neck, breast, and pelvic sites; and a total of 506 images obtained by electronic portal imaging device (EPID) were analyzed. The portal images acquired using the EPID systems attached to the Varian linear accelerators were superimposed on the reference images. The anatomy matching software (Varian portal Vision. 6.0) was used, and the displacements in two dimensions and rotation were noted for each treated field to study the patient setup errors. The percentages of mean deviations more than 3 mm in ‘lateral (X) and longitudinal (Y)’ directions were 17.5%, 11.25%, and 7.5% for breast, pelvis, and head and neck cases respectively. In all cases, the percentage of mean deviation with more than 5 mm error was 0.83%. The maximum average mean deviation in all the cases was 1.87. The average mean SD along X and Y directions in all the cases was less than 2.65. The results revealed that the ranges of setup errors are site specific and immobilization methods improve reproducibility. The observed variations were well within the limits. The study confirmed the accuracy and quality of treatments delivered to the patients.

## Introduction

Radiotherapy department at our oncology center was inaugurated in December 2004. This is the first audit study to verify the accuracy and reproducibility of treatments given to the patients. The aim of curative radiotherapy is to deliver a high dose of radiation to the tumor tissue; and at the same time, to keep the dose to the surrounding normal tissues to the minimum. Earlier studies have shown that the tumor control probability (TCP) has a close bearing on radiation beam placements and proper executions of treatment. Increasing the accuracy of radiation dose delivery to the intended target should improve the TCP and reduce treatment-related morbidity[1] Errors in patient positioning and beam placement were quantified by many studies,[2,3] and the need for various immobilization systems was emphasized.[4,5]

Electronic portal imaging (EPID) verification systems attached to treatment machines are able to study the accuracy and reproducibility of treatment executions[6] and document the delivered radiation dose.[7] Pretreatment patient positioning constitutes one important element in determining treatment accuracy. Currently, weekly port films are a standard method for assessing patient positioning accuracy.[8] Ravichandran *et al*.[9] have emphasized the need for field verification methods such as radiographic portal verification and the need for on-line imaging. Verhey and Bentel[10] have indicated a maximum shift of 3 mm with present-day immobilization methods; such shifts are random errors in the execution of daily treatments over a period of 5 to 6 weeks.

Comprehensive quality assurance (QA) program of a treatment is an important and essential aspect for the evaluation of tolerance limits and ensures adequate level of quality of treatment to the patients. The accuracy of the dose to the tumor and to the surrounding tissues and the precision in the spatial geometry of treatment volume are the main aspects of radiotherapy. The International Commission on Radiological Units and Measurements[11] recommended that the dose to the target volume should be delivered to within ±5%. With the advent of latest treatment delivery techniques with linear accelerators, recent clinical reviews recommend accuracies within ±3.5% in dose delivery.[12,13] World Health Organization[14] emphasized the need for QA for execution of radiotherapy, and Starkschall and Horton[15] outlined detailed procedures for implementation of QA.

The objective of the present study was to conduct an audit on QA aspects of treatment delivery, verification of the treatment field's position on different days, and to document the efficacy of immobilization methods and reproducibility of treatment fields on various orientations in beam-directed radiotherapy using EPIDs. The QA procedure with regard to treatment delivery was carried out in a retrospective study on 60 patients with 506 EPIs, which include 20 patients each treated for head and neck, breast, and pelvic sites. The mean and standard deviations were calculated and the results were analyzed.

## Materials and Methods

A QA procedure was formulated to study field placement errors, by comparison of reference images with the images taken by the EPID. The suggested procedure was implemented on patients treated using a high-energy linear accelerator, Clinac 2300 C/D with millennium multi-leaf collimator (MMLC-120) (Varian AG, USA); and low-energy linear accelerator, Clinac 600EX. Electronic portal images were acquired with an amorphous silicon detector EPID (aS500, Varian) and liquid ion chamber EPID (LC250, Varian) attached to the high- and low-energy linear accelerators respectively. The anatomy-matching software (Varian portal Vision.6.0) was used to study the patient setup deviations. The planned treatment parameters confirmed by the RT simulator (Acuity, Varian) and the Eclipse TPS (Varian) were checked using the RT information system (Varis) networked to the linacs.

Initially a portal image of active setup field of the patient was obtained prior to the treatment. The surface of the detector element of the imager was positioned at 140 cm from the source, and a double-exposure EPI was obtained. The weekly EPID images of each field used for the treatment of each patient were recorded during the course of treatment, the period of which varied from 4 to 6 weeks.

The field aperture contour was created on the reference DRR/simulator image. Then, the bony structures were drawn on the reference image using drawing tools. The portal image was superimposed on the reference image, and the bony landmarks on both the images were matched. The displacements along the X, Y directions and the rotation of the treatment field were recorded in a prescribed form, using the Varian portal vision anatomy-matching software tools on EPID. We measured the shifts in the AP/PA fields for pelvis, left lateral/right lateral fields for head and neck, and two tangential fields for breast cases. In all these cases, the fields were opposite to each other. In pelvis cases, the X and Y direction errors represent lateral (MI) and longitudinal (SI) shifts, whereas the X and Y direction errors in head and neck and breast cases represent vertical (AP) and longitudinal (SI) shifts respectively. Since the aim of the study was to determine the field setup errors and since parallel opposite fields were used in all the sites, the displacement errors are shown in ‘X’ direction and ‘Y’ direction.

In order to minimize subjective errors, the EPIs were analyzed independently by two observers and the deviations noted. Deviations less than 1mm between the readings of two observers were ignored and those more than 1 mm were corrected by taking the average of two sets of readings. The final values of deviations in all directions were noted. The mean and standard deviations were calculated and compared with the results of earlier studies. As discussed in literature,[16,17] it was assumed that the mean and standard deviations represent the systematic and random errors respectively. To represent true magnitude of errors, the positive and negative signs of the deviations were not considered in the calculations.

## Results

**Figure 1:**
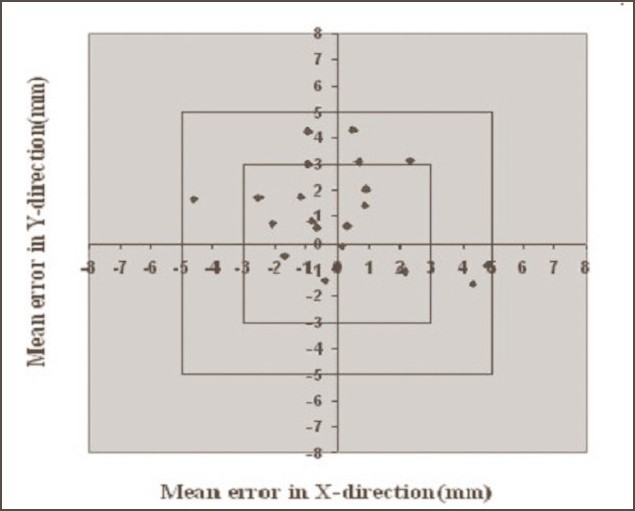
Systematic errors in breast cases without Orfit. Graph shows the number of average mean deviations <3 mm, between 3 and 5 mm, and >5 mm in X and Y directions

**Figure 2:**
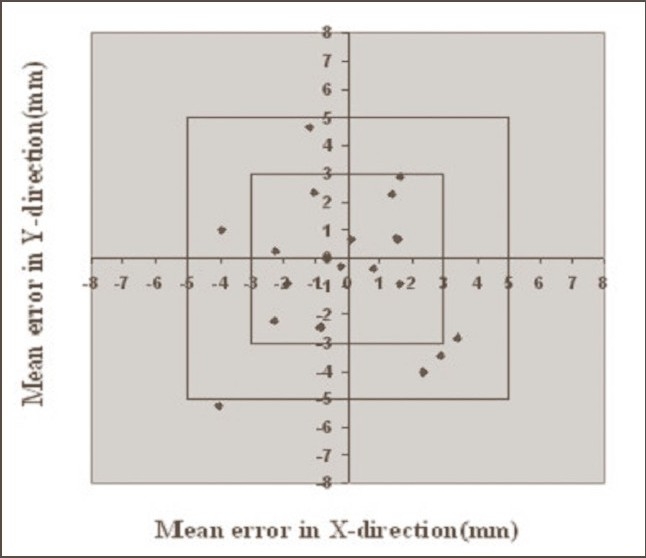
Systematic errors in breast cases with Orfit. Graph shows the number of average mean deviations <3 mm, between 3 and 5 mm, and >5 mm in X and Y directions

**Figure 3:**
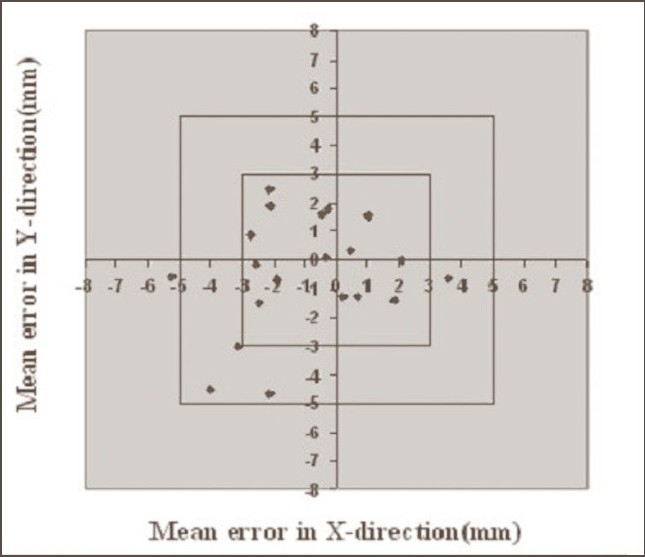
Systematic errors in pelvis cases without Orfit. Graph shows the number of average mean deviations <3 mm, between 3 and 5 mm, and >5 mm in X and Y directions

**Figure 4:**
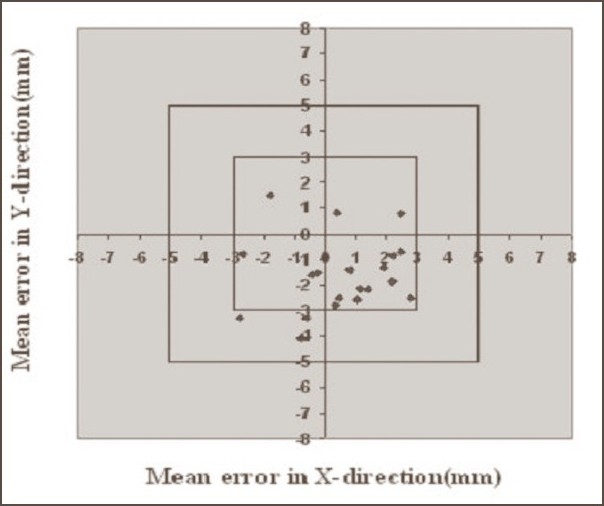
Systematic errors in pelvis cases with Orfit. Graph shows the number of average mean deviations <3 mm, between 3 and 5 mm, and >5 mm in X and Y directions

**Figure 5:**
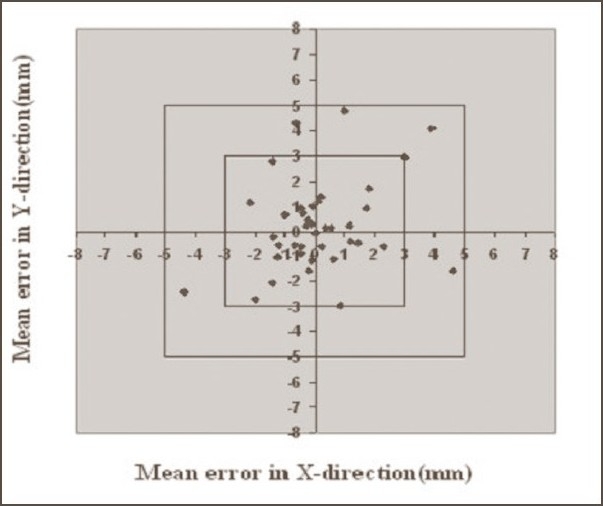
Systematic errors in head and neck cases with Orfit. Graph shows the number of average mean deviations <3 mm, between 3 and 5 mm, and >5 mm in X and Y directions

The data of deviations recorded from the verification of 506 EPIs was analyzed by a statistical method. The calculated mean and standard deviations for breast, pelvis, and head and neck cases, along with the study details, are shown in Table 1. The graphs of mean errors (systematic errors) in X and Y directions for breast and pelvis cases without and with Orfit are shown in Figures 1–4. The graph of mean errors for head and neck cases with Orfit is shown in Figure 5.

**Table 1:** Details of the study, along with calculated errors

*Site*	*No. of patients/No. of fields*	*No. of EPIs*	*% of mean errors >3 mm in X and Y direction*		*Maximum mean Setup error in X and Y direction (mm)*		*Average mean Setup Error in X and Y direction (mm)*		*Average mean SD Error in X and Y direction (mm)*	
			
			X	Y	X	Y	X	Y	X	Y
Breast cases without Orfit	10/20	93	15	20	4.86	4.3	1.64	1.74	2.08	2.20
Breast cases with Orfit	10/20	84	15	20	4.02	5.22	1.79	1.92	1.84	2.54
Pelvis cases without Orfit	10/20	83	20	10	5.23	4.65	1.55	1.93	1.58	2.65
Pelvis cases with Orfit	10/20	104	5	10	3.27	4.12	1.51	1.87	1.59	1.66
H and N cases with Orfit	20/40	142	5	10	4.63	4.80	1.17	1.29	1.07	1.48

The average percentages of cases in which mean deviations exceeded 3 mm in X and Y directions were 17.5%, 11.25%, and 7.5% for breast, pelvis, and head and neck cases respectively. In all cases, the maximum mean deviation did not exceed 5.23 mm; the mean deviation more than 5 mm being only 0.83%. The average mean errors (systematic errors) along X and Y directions were 1.71 and 1.83mm respectively in breast cases, where as the similar errors were 1.53 and 1.90mm for pelvis cases. The average mean errors along X and Y directions for the head and neck cases were 1.17 and 1.29 mm respectively. Similarly the SD (random errors) values of average means along X and Y directions were 1.96 and 2.37 mm for breast cases; 1.59 and 2.15 mm for pelvis cases; and 1.07 and 1.48 mm for head and neck cases.

## Discussion

The numbers of mean deviations more than 3 mm in X and Y directions in breast cases with and without Orfit were the same, and the ranges of mean deviations and standard deviations were comparable in both the cases. The number of mean errors larger than 3 mm were reduced by 50% with Orfit compared to that without Orfit in the pelvis cases. Similarly the ranges of mean deviations were less in Orfit cases, with an average mean of 1.69; compared to the average mean value of 1.74 in cases without Orfit. The numbers of systematic and random errors more than 3 mm were very low in head and neck cases. The errors were found to be reduced considerably in head and neck cases when compared with both the breast and pelvis cases. The ranges of mean deviations and standard deviations were also very small in head and neck cases compared to the breast and pelvis cases, as shown in Table 1.

It was observed that in all the cases, the mean and SD errors were greater in ‘Y’ direction compared to those in the ‘X’ direction, as shown in Table 1. We assume that this may be due to the lack of accuracy accepted on comparison of bony landmarks of matched images along the longitudinal (SI) direction i.e., Y direction compared to that along the lateral (MI) and vertical ie., (AP) directions i.e., X direction during the setup verification of fields. The increase in the number of errors in breast cases compared to the pelvis and head and neck cases may be due to the complex nature of breast setup geometry. The minimum number of errors obtained in the head and neck cases can be attributed to the use of Orfit immobilization and expected minimal distortions in the setup geometry due to the rigid nature of the site.

Hurkmans *et al*.[18] reported in their review article that the setup accuracy varies widely, depending on the treatment site, method of immobilization and institution. They reported that the standard deviation (SD) of systematic errors ranges from 1.6 to 4.6 mm for head and neck, 1.1 to 4.7 mm for pelvis, and 1.0 to 4.7 mm for breast cases. They further mentioned that by following the recommended procedures, the systematic and random setup errors that can be achieved in routine clinical practice can be less than 2.0 mm (SD) for head and neck; 3.0 mm (SD), for general pelvis. All our results are well within the above recommended limits and comparable to the sitewise trends observed by other workers, as mentioned in the above review article.

In all the cases, 80% of the rotational errors were less than 1°, 95% of the errors were less than 2°, and only 5% of the errors were within the range of 2° to 3°. Since the range and magnitude of all the rotational errors were very low, their effects were negligible and hence not discussed.

The retrospective study has revealed the probable range of systematic and random errors that occur in the site-specific field setup of patients during the course of radiotherapy treatment. The above study has built up a documented database for expressing the accuracy achieved by treatments delivered to the patients. The results obtained in the present study revealed that the maximum range of deviations was found decreased when compared to earlier results.[19] This can be attributed to the use of custom-made breast boards and Orfit immobilization casts, which have been used in the present study.

## Conclusion

The retrospective study has shown the probable range of systematic and random errors that occur in the field setup during the course of radiotherapy treatment. This work helped us to know the efficiency of immobilization methods in the reproducibility of patient position and feasibility of the field setup verification with the EPID. The results revealed that the ranges of setup errors are site specific, and the Orfit casts help to reduce the errors. This study confirmed that the observed variations were well within the limits of international standards. The study has also confirmed the accuracy and quality of treatments delivered at the National Oncology Center.
